# Supplementary material for: Host Lifeform Shapes Phyllospheric Microbiome Assembly in Mountain Lake: Deterministic Selection and Stochastic Colonization Dynamics
Source: Microorganisms. 2025 Apr 23;13(5):960. doi: 10.3390/microorganisms13050960 (PMC12113992; doi:10.3390/microorganisms13050960)
Supplement: Supplementary file 1 [file microorganisms-13-00960-s001.zip › microorganisms-3579220-supplementary.pdf]

Supplementary Materials

Host Lifeform Shapes Phyllospheric Microbiome Assembly in Mountain Lake: Deterministic Selection and Stochastic Colonization Dynamics

Qishan Xue [1], Jinxian Liu [1,2,*], Yirui Cao [1] and Yuqi Wei [1,2]

[1] College of Environment and Resources, Shanxi University, Taiyuan 030006, China

[2] Institute of Loess Plateau, Shanxi University; Shanxi Key Laboratory of Ecological Restoration on the Loess Plateau, Taiyuan 030006, China

Corresponding Author

Jinxian Liu *

Email: liujinxian@sxu.edu.cn

This file includes:

Supplementary Figure S1

Supplementary Figures

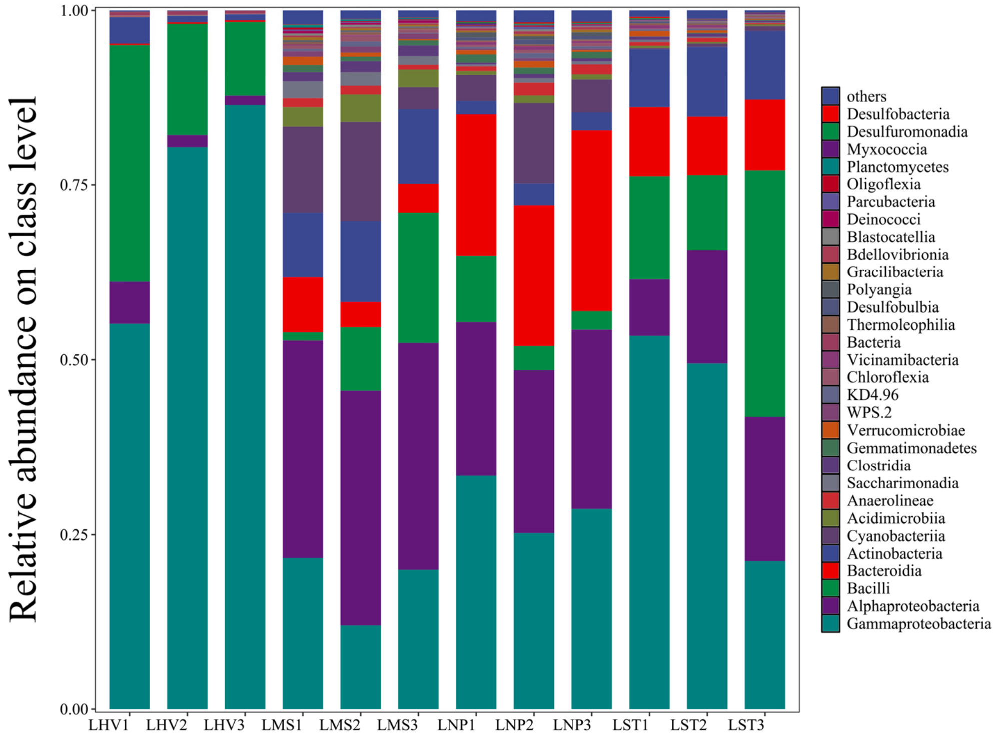

Figure S1. The composition of bacterial communities epiphytic on the leaves of four aquatic plants: (A) Dominant bacterial class. LHV refers to the leaves of *Hippuris vulgaris*, LMS refers to the leaves of *Myriophyllum spicatum,* LNP refers to the leaves of *Nymphoides peltatum*, and LSV refers to the leaves of *Scirpus validus*. The same applies hereinafter.
